# An Android-Based Heart Monitoring System for the Elderly and for Patients with Heart Disease

**DOI:** 10.1155/2014/625156

**Published:** 2014-08-19

**Authors:** Paola Pierleoni, Luca Pernini, Alberto Belli, Lorenzo Palma

**Affiliations:** Dipartimento di Ingegneria dell'Informazione, Universitá Politecnica delle Marche, Via Brecce Bianche, 60131 Ancona, Italy

## Abstract

The current trend in health monitoring systems is to move from the hospital to portable personal devices. This work shows how consumer devices like heart rate monitors can be used not only for applications in sports, but also for medical research and diagnostic purposes. The goal pursued by our group was to develop a simple, accurate, and inexpensive system that would use a few pieces of data acquired by the heart rate monitor and process them on a smartphone to (i) provide detailed test reports about the user's health state; (ii) store report records; (iii) generate emergency calls or SMSs; and (iv) connect to a remote telemedicine portal to relay the data to an online database. The system developed by our team uses sophisticated algorithms to detect stress states, detect and classify arrhythmia events, and calculate energy consumption. It is suitable for use by elderly subjects and by patients with heart disease (e.g., those recovering from myocardial infarction) or neurological conditions such as Parkinson's disease. Easy, immediate, and economical remote health control can therefore be achieved without the need for expensive hospital equipment, using only portable consumer devices.

## 1. Introduction

Recent technological advances in electronic and biomedical engineering are making it possible to move health monitoring away from the hospital and the clinic to portable personal devices [1]. Smartphones and biometric sensors are now available to all. These devices, which are often used in sport-related applications, are gaining an important role in health research and monitoring, enabling fast measurement of health parameters, accurate diagnosis of a number of conditions, and a reduction in hospital admissions.

Monitoring of heart function, accurate diagnosis of cardiac conditions, and prevention of heart disease are important goals. The electrocardiogram (ECG) is the gold standard method to detect cardiovascular abnormalities. It is a noninvasive diagnostic technique that records the electrical activity of the heart and enables diagnosis of a number of abnormalities and conditions based on a few simple features. The system described herein does not use a multichannel electrocardiograph but a much less expensive heart rate (HR) monitor and provides three types of tests:

- stress test, based on HR variability (HRV) and instantaneous HR;
- arrhythmia classification, based on R peak to R peak (RR) intervals;
- energy consumption evaluation, based on resting HR and instantaneous HR.

HRV can be related to chronic and acute stress [2]. Reduced HRV has consistently been documented in patients with anxiety disorders and chronic or posttraumatic stress, irrespective of individual features [3], due to autonomic nervous system alterations. Several studies have demonstrated connections between a chronic stress state and the risk of cardiovascular disease (CVD) [4]. Fast and easy testing of the main parameters of heart activity is therefore quite useful in daily life. A real-time personal stress monitor can provide continuous information on the user's stress level at all times and in any place.

RR intervals are important measures in sinus rhythm classification and identification of arrhythmia episodes and allow detection of sinus arrhythmia, premature ventricular contraction, ventricular tachycardia, ventricular flutter or fibrillation, and second-degree atrioventricular block [5]. A portable system providing hourly testing of heart activity and detecting episodes of arrhythmia would be valuable for patients with CVD. Several techniques are capable of providing these results; some detect a single type of arrhythmia, whereas others detect multiple types. The former techniques include time-domain analysis, sequential hypothesis testing algorithm, threshold-crossing intervals, artificial neural networks, time-frequency analysis, fuzzy adaptive resonance theory mapping, and sequential detection algorithm; the latter approaches include multiway sequential hypothesis testing, wavelet analysis, artificial neural networks, complexity measure, multifractal analysis, wavelet analysis combined with radial basis function neural networks, and nonlinear dynamic modeling [5]. These methods rely on ECG signal analysis; however, the analysis is not always technically feasible due to signal noise and the high computational cost of real-time analysis. Moreover such systems detect only a small number of arrhythmia types. We describe a validated method that allows real-time classification of sinus rhythm type and detection and classification of different arrhythmia events by an approach based on RR intervals and instantaneous HR [5].

Instantaneous HR is an important index of recovery after myocardial infarction and of the effect of medication for a number of neurological diseases [6, 7]. It can also be used to estimate the calories burned by any type of activity. Currently, the most accurate methods for measuring energy expenditure are based on calorimeters and respirometers, but they are quite expensive and are not suitable for portable devices. The portability requirement is met by approaches based on the HR monitor and the accelerometer, which provide effective means to estimate individual's energy expenditure during a given activity. We describe a novel HR-based algorithm that despite being less accurate than the methods mentioned above is sufficient for the application, which provides only an index of an individual's level of activity.

A number of similar systems and applications have already been devised, but none integrates powerful algorithms that process HR and the tachogram into an easy-to-use application that provides clear and immediate test reports to the user and to medical personnel. Moreover most existing systems merely display the raw data acquired by one or more wearable sensors [8], whereas others use sensors in the smartphone to show the raw data [9].

Another remarkable and innovative feature of the device described herein is that, unlike existing systems, which contain a single type of signal processing algorithm, it provides three different tests [10], that is, stress test, real-time classification of sinus rhythm type, and detection and classification of different arrhythmia events. This feature allows documenting correlations between stressful situations and arrhythmia episodes and enables CVD patients, who are prescribed exercise, to measure real-time calorie consumption. The test reports can then be sent to and stored in an online database that can be searched by a physician.

This paper shows how data about an individual's health state can be collected by real-time sampling and analysis of a few parameters using noninvasive, inexpensive, and portable devices.

## 2. Materials and Methods

**Figure 1:**
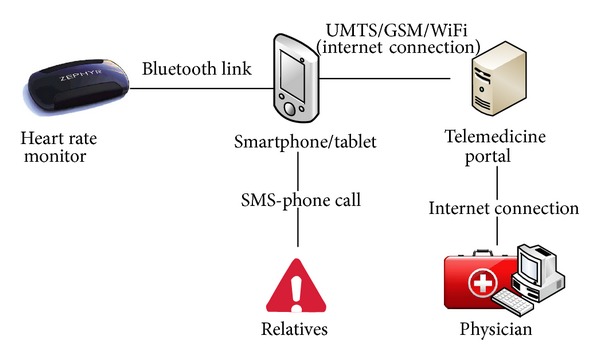
System architecture.

The HR monitor used in this work is a Zephyr HxM-BT device (Zephyr Technology Corporation) that was chosen for its modest cost, high usability, and good performances. An application for Android OS, which is widely applied in portable devices all over the world, was developed by our team. Its main tasks include data processing; reporting of test results to the user; test scheduling during the day; dialing of automatic emergency calls or sending an SMS in case of critical user conditions; and data storage in a telemedicine portal database (Figure 1). The application also provides the controls, settings, views, and Bluetooth connection management required for easy system use and clear test reporting.

### 2.1. Data Acquisition and Storage

A Bluetooth connection between a smartphone or a tablet and the HR monitor provides secure data acquisition. The HR monitor used in this work transmits data packets containing information acquired by biosensors at a frequency of 1 Hz [11]; a routine implemented in the Android application extracts from each packet the following information: instantaneous HR, last 15 heartbeat time stamps, and last heartbeat progressive number. Unless a test is in progress the smartphone display shows only the HR. The test results are then saved to an internal SQLite database and may be sent to an online MySql database via Wi-Fi or a 3G connection of the Android device using Json format and Php web pages.

### 2.2. Stress Test

HRV analysis in the time domain is based on the calculation of a number of statistical indices over an appropriate data acquisition window [12]. A first key feature that needs to be calculated is the standard deviation of the RR interval (SDNN): this is the square root of variance and is related to all the cyclical components connected to the variability detected during the observation period. It is important to note that HRV variance depends on the length of the observation period; measurement repeatability and comparability require this period to be established in advance [13]. A standard duration (e.g., 5 minutes) therefore needs to be set. The next feature to be extracted is the square root of the mean squared differences of successive RR intervals (rMSSD). The last collected feature is the proportion that is obtained by dividing the number of interval differences of successive RR intervals greater than 50 milliseconds (ms) by the total number of RR intervals, in the observation window (pNN50). These data and the average HR (HR_AVG_) are the key stress test parameters and are based on a decision threshold for each feature.

Since in the case of stress testing the analysis requires the heartbeat time stamps, the algorithm stores these values: a number of R peak time stamps are extracted from each packet depending on the heartbeats that have been detected after the latest packet has been received. This control is enabled by checking the heartbeat number extracted from each packet. Then the RR intervals are calculated in real time from the time stamps and stored.

**Figure 2:**
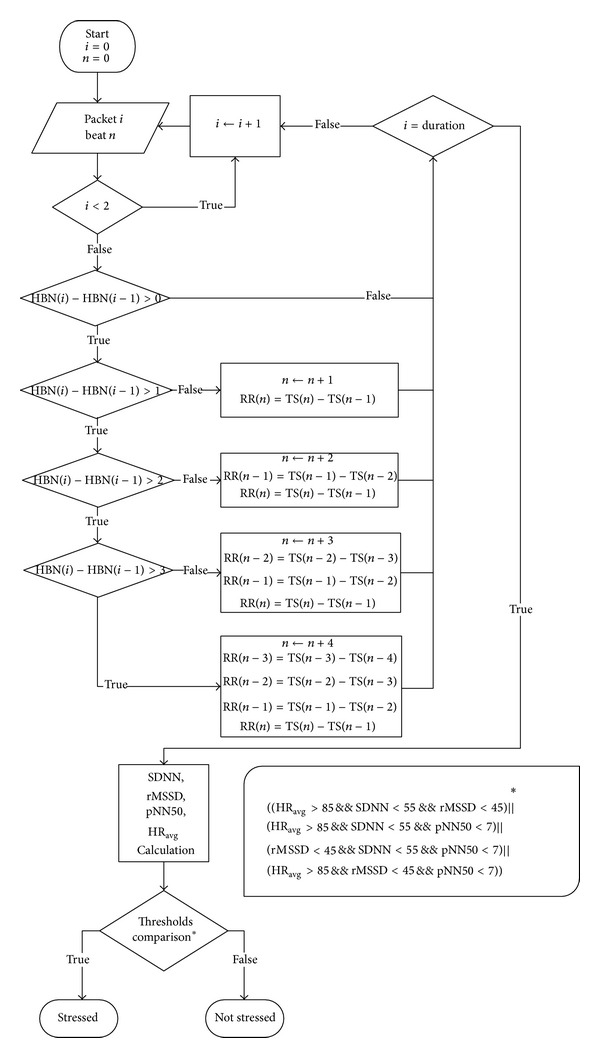
Stress test algorithm block diagram.

At the beginning of the algorithm (Figure 2), there are *i* = 0 packet received and *n* = 0 heartbeats detected. After the arrival of the *i*th packet, the first control is whether the index is <2. If this is true the system must wait for the next packet before processing the data; if it is false the control is on the heartbeat number (HBN). If the difference from last HBN to previous HBN is 0, no new heartbeats have been detected and the algorithm waits for the next packet. If the difference is 1, 2, 3, or 4, new heartbeats have been detected and the relevant RR intervals are calculated and stored. The limit is 4 heartbeats because the maximum HR measurable by the HR monitor is 240 beats/minute or 4/second. The next control involves index *i* which is compared with the predetermined duration of the analysis. In fact, this index is also used as the timer of the algorithm in relation to the frequency of 1 packet received/second. If the index is less than the duration of the observation window the system waits for the next data packet before repeating the steps; otherwise, it calculates all the statistical features and compares them with the threshold, to determine whether a stress state is present. The decision is reached by majority vote: if three of the four values exceed (HR_AVG_) or are lower than (other features) the thresholds (Table 1) [14], the stress state is considered to be present [15].

It is important to note that SDNN is calculated at 5-minute intervals regardless of the duration of the analysis and then averaged over that duration, because this parameter changes with the length of the interval and because the relevant threshold is referred to as a 5-minute window [13]. The evolution of the tachogram is plotted on the phone display and the test results are finally shown and stored in a local database. The application also allows alerting a distant person (e.g., a relative) by dialing an emergency call with a prerecorded message or sending an SMS to a favorite number if a stress state is detected. The test results can also be stored in the patient's account in a telemedicine database. The real-time stress analysis algorithm, which includes the formulas and the HRV thresholds mentioned above, has been wholly developed by our research team.

### 2.3. Detection of Episodes of Arrhythmia

Arrhythmia may occur in a healthy heart with minimal consequences. It may present as respiratory sinus arrhythmia, a natural periodic variation in HR, or it may be an index of a severe heart condition [16]. An automatic device providing real-time arrhythmia detection and rhythm classification therefore has the potential to be highly useful in the daily monitoring of patients with heart conditions.

The method described herein provides sinus rhythm type classification and detection and classification of different arrhythmia events using an approach based on RR intervals and instantaneous HR [5]. It is based on three algorithms running in parallel.

#### 2.3.1. Sinus Rhythm Classification

The most immediate analysis provided by the system is classification of the supraventricular rhythm as normal, bradycardia, or tachycardia.

**Figure 3:**
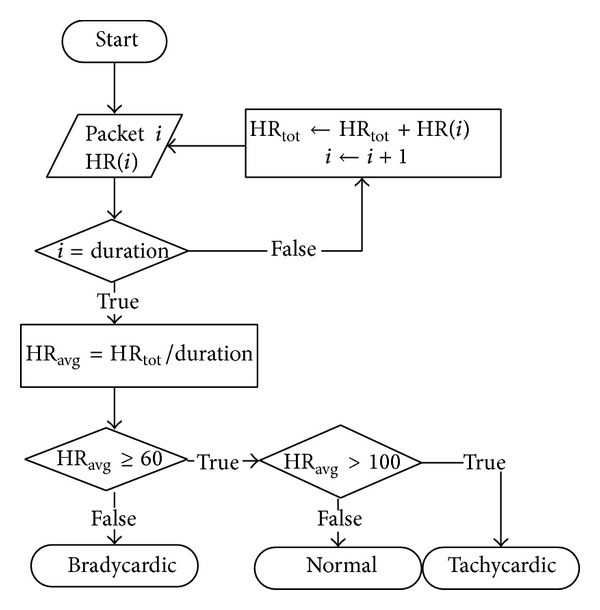
Sinus rhythm classification block diagram.

The algorithm calculates the HR_AVG_ during the observation window (Figure 3), with the user at rest. It extracts the instantaneous HR of each packet and adds up to a total value HR_TOT_. The HR_AVG_ is then obtained by dividing the HR_TOT_ by the duration of the observation window, that is, the total number of packets received, and is compared with two thresholds: the rhythm is classified as bradycardia if HR_AVG_ is <60 beats per minute (bpm), normal if is comprised between 60 and 100 bpm, and tachycardia if is >100 bpm (Table 2) [17].

#### 2.3.2. Sinus Arrhythmia Detection

Sinus arrhythmia is also detected by our system. It is a common supraventricular rhythm and is related to breathing: HR increases during inspiration (Bainbridge reflex) and decreases during expiration (vagal effect). It affects especially young people, where it is not considered pathological.

**Figure 4:**
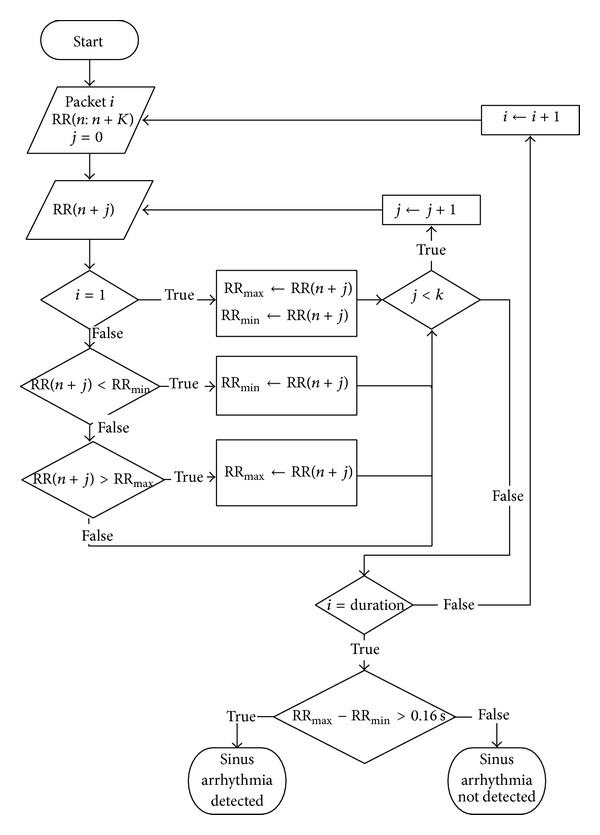
Sinus arrhythmia detection block diagram.

Our algorithm uses RR intervals (Figure 4): since each packet contains *k* < 4 new beats, the relative RR intervals are calculated and compared with the actual RR_MAX_ and RR_MIN_ values and if necessary replaced with them. When the total number of packets received matches the predetermined observation window, the difference between the longest and the shortest RR intervals is calculated to check if it is >0.16 seconds (s) [18]. If this is true, sinus arrhythmia is considered to be present and a report is generated on the phone display.

The sinus rhythm classification and sinus arrhythmia detection algorithms have been wholly developed by our team for real-time use.

#### 2.3.3. Detection of Other Arrhythmia Types

Other types of arrhythmia can be detected and classified using a more complex algorithm. The method implemented in our device involves two steps: arrhythmia beat classification and arrhythmia episode classification. The episodes that can be classified include ventricular premature systole, ventricular bigeminy, ventricular trigeminy, ventricular couplet, ventricular tachycardia, ventricular flutter/fibrillation, and 2nd degree heart block.

**Figure 5:**
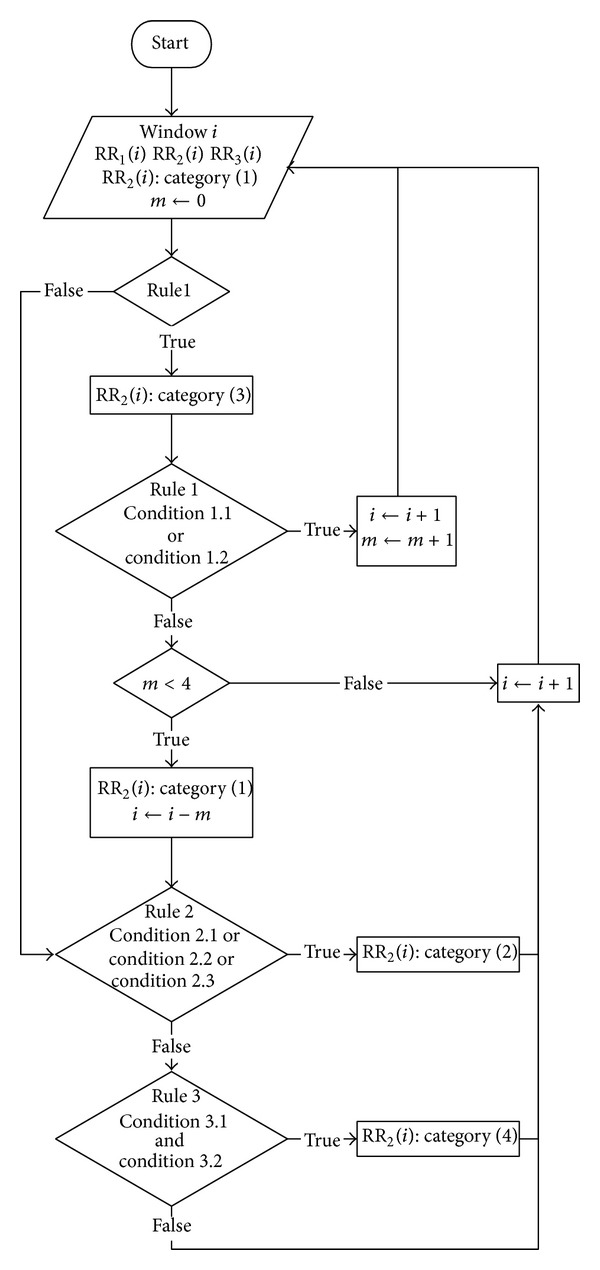
Arrhythmia beat classification block diagram.

This algorithm also uses RR intervals. It is based on the algorithm described by Tsipouras et al. [5]; we improved it adding real-time implementation. The first step is beat classification (Figure 5). This preliminary task is based on a 3-RR interval window (RR_1_, RR_2_, and RR_3_) that slides one beat at a time, where the RR interval to be classified is the middle one. This beat is initially considered as normal and may subsequently be assigned to another category based on three rules related to medical-clinical data. There are four categories: (1) normal sinus beat (N); (2) premature ventricular contraction (PVC); (3) ventricular flutter/fibrillation (VF); and (4) 2nd degree heart block (BII). These acronyms are used in the MIT-BIH database. The three rules are as follows.

*Rule 1*. VF: it is activated if RR_1_ > 1.8RR_2_ and if the duration of RR_2_ is less than 0.6 s. In this case RR_2_ is considered as the beginning of a VF episode and subsequent windows are tested for the following two conditions:

- Condition 1.1: the duration of each RR-interval in the window is <0.7 s;
- Condition 1.2: the overall duration of the RR_1_ + RR_2_ + RR_3_ window is <1.7 s;

If one of the two conditions is met the middle RR interval is classified as category (3). The rule is based on classification of the whole VF episode, not a single beat, and is applied to *m* consecutive windows. A VF episode will be detected only if the *m* index of windows containing beats classified as category (3) is ≥4. The algorithm continues with the next rule, coming back to the *i* − *m* window if neither condition is true for at least 4 consecutive windows or if Rule 1 is not verified.

*Rule 2*. PVC, category (2): this state is present if one of the following conditions is true:

- Condition 2.1: if RR_1_ > 1.15RR_2_ and RR_3_ > 1.15RR_2_, the RR_2_ interval is classified as an isolated PVC;
- Condition 2.2: if |RR_1_ − RR_2_| < 0.3 s and both RR_1_ and RR_2_ are <0.8 s and 1.2(RR_1_ + RR_2_)/2 < RR_3_, then we have a PVC couplet;
- Condition 2.3: if |RR_2_ − RR_3_| < 0.3 s and both RR_1_ and RR_2_ are less than 0.8 s and 1.2(RR_2_ + RR_3_)/2 < RR_1_, then we have a PVC couplet.

*Rule 3*. BII, category (4): if conditions 3.1 and 3.2 are both true, the RR_2_ interval is assigned to this category:

- Condition 3.1: 2.2 s < RR_2_ < 3.0 s;
- Condition 3.2: |RR_1_ − RR_2_| < 0.2 s or |RR_2_ − RR_3_| < 0.2 s.

The algorithm is repeated for all subsequent windows. The rules are mutually exclusive and must be applied in the reported sequence. After a beat has been classified by one rule its classification cannot be changed by another.

**Figure 6:**
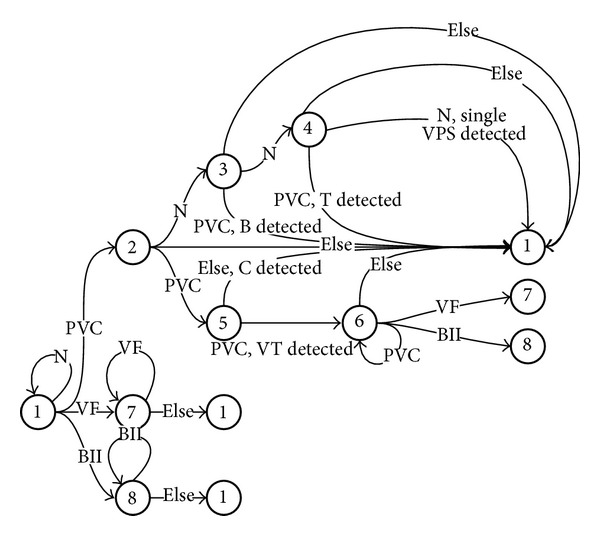
Arrhythmic episode classification by the automaton.

The results of beat classification are used as input of the automaton (Figure 6) for detection and classification of the arrhythmia episode. The rhythm classification categories and relevant MIT-BIH symbols are normal sinus rhythm N, ventricular premature systole VPS, ventricular bigeminy B, ventricular trigeminy T, ventricular couplet C, ventricular tachycardia VT, ventricular flutter/fibrillation VFL, and 2nd degree heart block BII. The default rhythm is normal, unless an episode of one of the above rhythms is detected. Episodes start with a distinctive beat—for instance, a PVC beat is the first beat for B, T, and VT episodes; VF for VFL episodes; and BII for BII episodes—and are characterized by a specific sequence pattern; they then end with a nonspecific beat. B has a PVC-N-PVC-N-⋯-N-PVC pattern, T has a PVC-N-N-PVC-N-N-⋯-PVC-N-N-PVC pattern, the pattern of VC is PVC-PVC-not PVC, and the pattern of VT is PVC-PVC-PVC-⋯-VC structure (≥3 consecutive PVCs). A major challenge to be met by the automaton is discrimination among these arrhythmia types, due to the similarity of the initial phase of each episode. VF episodes start with a VF beat and the number of consecutive VF beats determines its duration. The same applies to BII episodes. The states of the automaton are as follows.

- State 1: start of the automaton: the automaton remains in this state as long as the input is an N beat. It moves to states 2, 7, or 8 if a PVC, a VF, or a BII beat occurs, respectively.
- State 2: possible B, T, and C and single VPS or VT: the automaton goes to state 3 if the next beat is N; to state 5 if the next beat is a PVC; or to state 1 if the next beat is neither an N nor a PVC.
- State 3: possible B and T and single VPS: if the next beat is a PVC and B is detected the automaton returns to state 1. If the next beat is N, it moves to state 4; if the beat is none of the above types, it moves to 1 and no episode is detected.
- State 4: possible T and single VPS: if the next beat is a PVC, a T is detected, whereas if the beat is N, a single VPS is detected; otherwise, nothing is detected and the automaton returns to state 1.
- State 5: possible C and VT: if the next beat is not a PVC, C is detected and the automaton returns to state 1; if it is a PVC, the automaton moves to state 6.
- State 6: VT is detected: if the beat is a PVC, the automaton remains in state 6 until the VT episode has ended. If the next beat is a VF or a BII, the automaton moves, respectively, to state 7 or 8; if it is different, it moves to state 1.
- State 7: VFL is detected: if the next beat is a VF, the automaton remains in state 7 and the episode is considered to continue. In any other case the automaton returns to state 1.
- State 8: BII is detected: if the next beat is a BII, the automaton remains in state 8 and the episode is considered to continue. In any other case the automaton returns to state 1.

The automaton returns to state 1, and the previous episode is considered as completed (and is reported by the system if it has reached the minimum required HBN); if an unexpected beat occurs, the new episode is ignored.

### 2.4. Evaluation of Energy Consumption

The approach described herein requires knowledge of the user's weight, height, gender, age, and resting HR. Calorie consumption is then calculated by integrating the HR value over the observation period. The algorithm used in our application is evolution of the algorithm developed by Lee and coworkers [19] and exploits the computational abilities of current smartphones: HR is continuously monitored instead of being sampled at predetermined time points. Real-time analysis is the main improvement over the previous method. Notably, the new approach can be extended to any type of physical activity.

The method developed by Lee and coworkers requires entering resting HR, initial HR, final HR, and total duration of the analysis. This entails a number of limitations.

- The requirement of knowing total analysis duration and final HR precludes calculation in real time.
- It assumes that the final HR is the peak value in the entire exercise; any pauses or final cooling down may thus affect the calculation.

The previous method [19] makes integration to calculate energy consumption: three types of instantaneous energy consumptions are computed by functions that are linearly dependent on HR and are subsequently integrated over the total duration of the analysis. The method involves three calculation steps:

- calorie consumption at rest over the entire activity;
- calorie consumption after starting the exercise and up to the peak HR (10% of total duration);
- calorie consumption during maximum sustained HR (90% of total duration).

Therefore, pauses or interruptions of any type are not accommodated.

The method developed by our team analyzes 10-second windows and involves two steps:

- calculation of calorie consumption at rest;
- calculation of calorie consumption during exercise based on the mean HR sampled in the interval.

The two steps provide the final calorie consumption value, which is updated every 10 s by adding the two new values. The equation for estimating energy expenditure at rest (step (i)) is

(1) K1i[kcal]=t[(hr1−hr0+20.25)+C] ×(0.01808s+0.00895(1−s)),

where *K*_1__*i*_ is the energy consumption of the *i*th interval in kilocalories, hr_0_ is the HR at rest, hr_1_ is the HR at the beginning of analysis, *C* is a coefficient of basal metabolic rate (per second in this case), *s* is a value depending of gender (1 for male, 0 for female), and *t* is the interval duration (in s).

The basal metabolic rate allows calculating energy expenditure based on user physical features [20]:

(2) bmr=(655+9.6w+180h−4.7a)(1−s) +(66+13.7w+500h−6.8a)s,

where *w* is the user's weight (in kg), *h* is the height (in m), and *a* is the age (in years).

The equation calculating calorie consumption per second of exercise in steps (ii) and (iii) is

(3) Y[kcals]=[Bm(hrx−hr0)+C+0.3645]s +[Bf(hrx−hr0)+C+0.1812](1−s),

where hr_*x*_ is the HR at the start or at the end of exercise, whereas *B*_*m*_ and *B*_*f*_ are

(4) Bm=0.0109LBMh2−0.0023%FAT−0.0007a−0.0211,Bf=0.014LBMh2−0.0012%FAT−0.01254.

LBM is lean body mass, equal to

(5) $$\begin{array}{c} \text{LBM}=w\left(1-\%\text{FAT}\right). \end{array}$$

%FAT is the percentage body fat [21]:

(6) $$\begin{array}{c} \%\text{FAT}=\frac{\left(1.2\text{BMI}+0.23a-10.8s-5.4\right)}{100}. \end{array}$$

BMI is the body mass index [22]:

(7) $$\begin{array}{c} \text{BMI}=\frac{w}{h^{2}}. \end{array}$$

The calculation involved in step (iii) is as follows (step (ii) is not used in our method):

(8) $$\begin{array}{c} {K_{3}}_{i}=\left(Y\left(\text{h}{\text{r}}_{i}\right)-Y\left(\text{h}{\text{r}}_{s}\right)\right)t, \end{array}$$

where hr_*i*_ is the mean HR value in the *i*th interval and *t* is interval duration. *K*_3__*i*_ is also calculated for each interval.

Total *K*_1_ and *K*_3_ values are then added to obtain *K*_tot_:

(9) $$\begin{array}{c} K_{\text{tot}}=\overset{N}{\underset{i=0}{\sum }}\left({K_{1}}_{i}+{K_{3}}_{i}\right) \end{array}$$

assuming *N* as the windows number.

**Figure 7:**
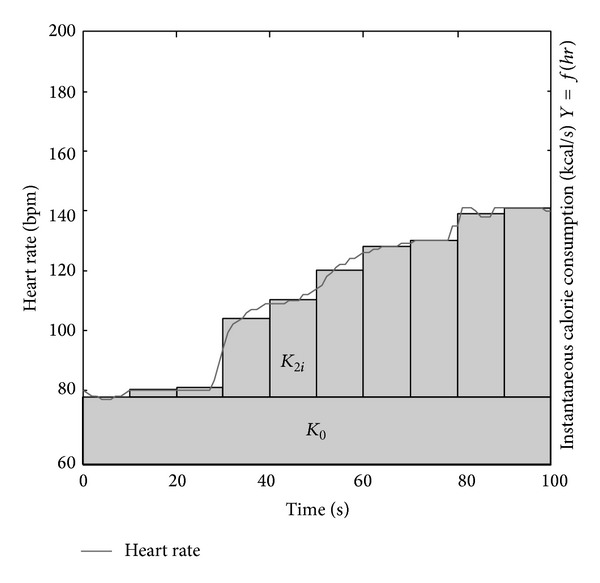
Calorie consumption calculation method.

Figure 7 shows the calculations described above. *K*_tot_ is the grey area and *K*_1_ is the sum of *K*_1__*i*_.

### 2.5. Application Details

The other functionalities developed by our team include a Bluetooth connection, graphical interfaces, user settings, and alarm management.

The Bluetooth connection between smartphone and HR monitor is established using Android Bluetooth APIs and the Java library of the pulse sensor [23]. The only requirement for the connection is pairing the two devices via the Android settings menu. After selection of a user command that prompts the appearance of a connection button on the screen, the application searches for the device, which is listed in the bonded device list, and a Bluetooth socket is established. A connection thread listens for incoming packets and saves them in a buffer. Finally, the required information is extracted from the packets and sent to the class assigned to the processing using the* obtainMessage* method (Handler class).

**Figure 8:**
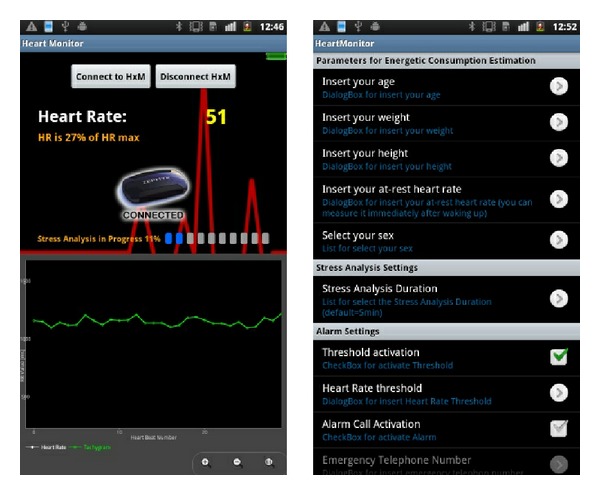
Application interfaces: main interface and settings interface.

The user can interact with the application through a main interface, a menu interface, and the settings interface (Figure 8). The main interface contains buttons for device connection and disconnection via Bluetooth and shows several items of information: the connection status, the battery status of the connected pulse sensor, the instantaneous HR, and the progress and results of current tests. It may also show the HR trend or the tachogram (RR interval trend); these charts are created using the* aChartEngine* library and implement zooming and scrolling functionalities.

The menu interface allows activating/disabling the different analysis algorithms, clearing the chart, accessing the settings menu, viewing data history, and quitting.

Using the Service class, a background part of the application reminds the user to perform any scheduled tests during the day.

The settings interface makes it possible to enter user features (age, weight, height, resting HR, and gender), set test duration, manage alert messages and calls (activation, phone number, and preconfigured text messages), and schedule tests.

Alerts are sent to a remote phone using* SmsManager* and* TelephonyManager*.

Finally HR, RR interval signals, and test reports can be saved to ^∗^.txt files and to a local SQLite database, ready to be sent in Json format to a telemedicine portal consisting of a MySql database and Php web pages.

## 3. Results and Discussion

### 3.1. Stress Test Results

**Figure 9:**
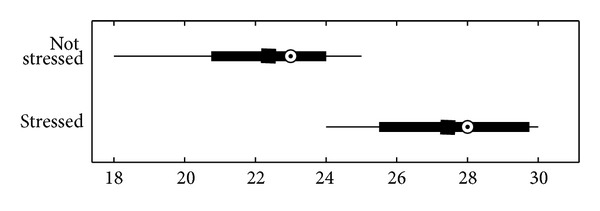
Statistical box-plot. *x*-axis: results of the PSS questionnaire; *y*-axis: participants divided by the algorithm into “stressed” and “nonstressed.”

The stress test was assessed in a trial involving 20 healthy individuals (6 female and 14 male university students aged 23–27 years without neurological disorders) who had abstained from coffee and alcoholic beverages for 12 hours. Participants underwent analysis by our application for 10 minutes and the test results were not shown. They subsequently completed the validated 10-item Perceived Stress Scale (PSS) questionnaire [24]. The test data and the results of the questionnaire are reported in Table 3, where subjects are divided into stressed and nonstressed based on test results and the mean PSS score is reported for the two groups. The same data are shown in Figure 9 in a traditional box-plot, where the dots are the median values and the squares the mean values.

The two datasets were assumed to have normal distribution. The resulting *t* value is 4.49, *P* = 0.0003 (Student's *t*-test). The mean PSS scores of the two groups were significantly different (*P* < 0.01).

### 3.2. Results of Arrhythmia Episode Detection

Sinus rhythm classification and sinus arrhythmia detection were tested in 25 healthy students aged 23–29 years. The experiment involved 10-minute parallel testing with our device and an ECG. The algorithm output simply described the subject's cardiac rhythm and whether he/she presented sinus arrhythmia. The ECG tracings were assessed by a cardiologist and compared to the test results reported by our application. The two sets of test reports matched, demonstrating that the algorithm is 100% accurate in sinus rhythm classification and sinus arrhythmia detection. As regards the sinus rhythm, 18 participants had a normal rhythm, 5 had a bradycardic rhythm, and 2 had a tachycardic rhythm. Moreover, 16/25 subjects presented sinus arrhythmia, which is common among the young. These data are reported in Table 4.

The detection and classification of arrhythmia events have previously been tested by Tsipouras and coworkers [5], who found very high sensitivity and specificity. We tested our real-time Java implementation by an offline simulation using as input the values available on the MIT-BIH database rather than the signal acquired from the heart monitor. We found 94% accuracy in detection and classification of arrhythmia episodes, the same result obtained by Tsipouras et al. [5].

### 3.3. Results of Energy Consumption Evaluation

The energy estimation algorithm described herein was compared with the method used by Garmin Ltd. This method was chosen as the reference because Garmin is widely considered a leader in its sector. In addition a large amount of data that can be used for comparisons is available on the Garmin Connect platform.

**Figure 10:**
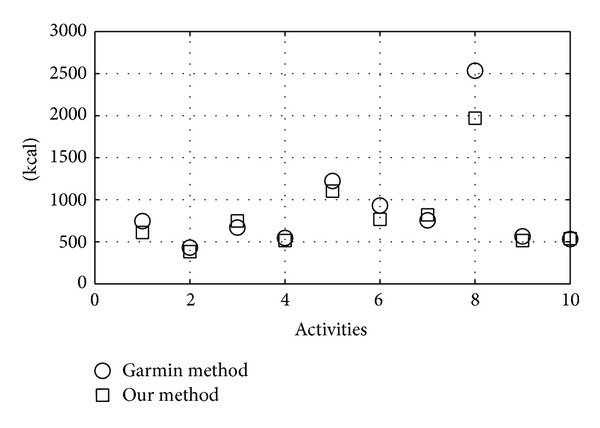
Comparison of calorie calculation data from the Garmin method and our algorithm.

For this test we used offline implementation of our algorithm that simulates real-time device operation. The algorithm was applied to 10 different HR acquisitions relating to 10 subjects and three different types of activity (available on the Garmin Connect web platform). The results are shown in Table 5. The results obtained by our device are very close to those obtained by Garmin's method (Figure 10). The Pearson linear correlation coefficient between the two datasets is *r* = 0.9865. Since the test used merely indicative information about user activity levels, the results are quite acceptable.

### 3.4. Application Usability and Usefulness

The final feature that needed to be tested was user perception. To do this we administered to the 20 participants involved in stress test validation an additional questionnaire, the 10-item System Usability Scale (SUS) [25], which is a platform-independent model widely used in subjective software evaluation. The mean values, converted values, and standard deviation (Std) for each question as well as total SUS scores are reported in Table 6. The total per cent score is 75.625, which corresponds to a B grade and is therefore a good result [26]. The Std values are quite small, entailing narrow spread around the mean. In more general and objective terms, users and patients who have used the application during the test expressed their satisfaction in the use. Importantly, prolonged use of the application did not evidence bugs or unexpected system crashes.

## 4. Conclusions

This paper describes a real-time heart monitoring system suitable for a variety of users including elderly individuals and patients with CVD. The system is based on a simple, inexpensive, and wearable device whose functionalities are comparable to those of more expensive and sophisticated clinical devices. The system's high-level performances are related to a set of complex algorithms that have been implemented in the Android device. The stress test, heart rhythm classification, and sinus rhythm detection algorithms have been developed entirely by our group, whereas the arrhythmia classification and detection algorithm and the algorithm evaluating energy expenditure employ known methods that were improved to suit the real-time and mobile conditions of the present application.

The stress test and the arrhythmia algorithms proved to be able to identify a stress state and episodes of arrhythmia. Clearly, the computer software cannot replace a physician, but it does provide information and alerts. The energy expenditure algorithm calculates calorie consumption in real time; it is based on an existing method that was improved by our team and extended to cover a number of general conditions (exercise with breaks, exercise with cooling down, and normal daily activities). These features make the device suitable for users who need to monitor calorie consumption during the day due to health reasons.

An additional novel feature of the device is that it integrates three different algorithms for HR and tachogram processing in a single mobile application that (i) provides remote monitoring of patients outside the hospital, (ii) is easy to use, and (iii) provides clear test reports to users and medical staff. All the data about the patient's health status can be stored in an online database.

The HRV and arrhythmia algorithms provide useful information about patients with a range of diseases. For example it has been reported that there may be a close association between reduced HRV and the severity of Parkinson's disease (PD) [27]. Indeed, PD affects the diurnal autonomic cardiovascular regulation, whose alteration seems to be more profound in patients with more severe disease. The result of these changes is a reduced HRV, but current knowledge of the long-term consequences of certain HR features and HRV, for example, circadian regulation, is limited. We are currently collecting heart activity data from PD patients at the Regional Hospital Department of Rehabilitation (Ancona, Italy) with a view both to validate our system and to test the hypothesis of a correlation between PD and abnormal HRV.

We are also testing the algorithms developed for our device and the scope for its application to a number of conditions and uses in view of its marketing in the near future.

## Figures and Tables

**Table 1:** Thresholds for detection of stress state.

Variable	Threshold	Unit
HR_AVG_	>85	Beats/min
pNN50	<7	%
SDNN	<55	ms
rMSSD	<45	ms

**Table 2:** Thresholds for supraventricular sinus rhythm classification.

Sinus rhythm type	HR (bpm)
Bradycardia	HR < 60
Normal	60 ≤ HR ≥ 100
Tachycardia	HR > 100

**Table 3:** Results of the stress test algorithm.

Condition	Number of subjects	PSS mean score	PSS variance
Not stressed	13	22.3	5.6
Stressed	7	27.6	4.7

**Table 4:** Results of the sinus rhythm classification and the sinus arrhythmia detection algorithm.

Sinus rhythm	Number of subjects	Confirmed by ECG
Normal	18	100%
Bradycardic	5	100%
Tachycardic	2	100%
Arrhythmia	16	100%

**Table 5:** Results of the energy expenditure algorithm.

Activity	Garmin method (kcal)	Our method (kcal)
Walking	745	610
Walking	430	382
Running	669	747
Running	545	515
Running	1222	1102
Running	930	766
Cycling	755	820
Cycling	2533	1968
Cycling	564	515
Cycling	532	537

**Table 6:** Results of the System Usability Scale (SUS) questionnaire.

Question	Mean value	SUS converted value	Std
Q1	3.75	2.75	0.79
Q2	2.2	2.8	0.95
Q3	4.2	3.2	0.7
Q4	1.75	3.25	0.79
Q5	3.35	2.35	0.81
Q6	1.95	3.05	0.89
Q7	4.6	3.6	0.6
Q8	2.3	2.7	0.66
Q9	4.05	3.05	0.83
Q10	1.5	3.5	0.6

Total score		% score	

30.25		75.625	
